# On the Application of the Collegiate System to the Medical Schools of London

**Published:** 1842-01

**Authors:** 


### On the Application of the Collegiate System to the Medical Schools of London.

Amid the loud and justifiable cries for medical reform with which the whole kingdom at present rings, it is both wholesome and desirable to receive the faithful warnings of a friend who calls upon us to look at home and to rectify evils which lie at our own door and concern us, as a profession, still more nearly. Into this category, unquestionably, must come those evils—great and manifold—which spring from the total deficiency of any system of collegiate discipline in our medical schools. The reform of these depends entirely, or for the most part, upon the conscientious sense of duty in the members of the profession itself; they demand no parliamentary interference; their removal will infringe the rights of no corporate bodies, while every step that shall be taken towards their correction will be in itself a positive good, a blessing conferred upon the rising members of the profession, and through them on the country at large. It is therefore with much satisfaction that we see the subject of collegiate discipline again brought prominently forward,* and we are disposed to hope, from the notice which has been taken of it in all our medical journals, that a strong feeling in favour of the cause it advocates is already excited. Most assuredly the man will well deserve of the country who, by a well-devised plan of moral superintendence, shall enable the medical student to escape the multitude of temptations which surround him just at the most critical period of his life, and instruct him in the best mode of occupying his short and valuable period of hospital studies.

We well know, from experience, the dismay with which the medical student looks around him upon the crowd of strangers who pass before him, on his first leaving the retirement of a country town for the busy mart of intellect in London. What would he not give to meet with some companion, sufficiently experienced, and sufficiently trustworthy to guide him in his choice of lectures, of books, of society? He looks at the list of lectures, of hospital attendances, of demonstrations, all of which he is required to attend, and comparing the space of time which he is to be allowed with the tasks to be performed in it, he is utterly at a loss and in despair. He has not yet learned to sift the essential from the non-essential, and is ignorant of the processes whereby even the industrious student is obliged to lighten his labour, and by which the idler contrives to discover the minimum of study required for his passing the *pons asinorum* of the Hall and College examinations. He comes up to the schools, probably, with the most upright and conscientious intentions of learning his profession, and of redeeming that time which has inevitably been too much wasted in a five years' apprenticeship. He comes to the task in all the ardour of youth, with the most sanguine anticipations of success; but he is utterly bewildered and disheartened by the multiplicity of demands upon him. He looks around for some friendly hand to guide him in his difficulties, and the chance is at least an even one, that, deceived by an assured air of importance and pretension, he is led into society the very reverse of what is desirable for an inexperienced youth, and the influence of which may greatly prejudice and mar the whole progress of his future studies and of his future life.

But the want of a judicious system of discipline is not less felt by the student in the subsequent part of his career. He has no friendly tutor to encourage him in his studies, to aid him with his advice, to warn him against evil companions. There is no check either upon his time or his money. He is surrounded with temptations, and has nothing but his own good sense and good feeling, (that of an inexperienced youth of seventeen,) to preserve him from them. His studies are to be directed solely by his own judgment; and as for religion—as if a matter apparently of the least consequence of all—there is no provision either for his instruction in it, or for his attendance upon its ordinances.

* A Letter to Sir B. Brodie, Bart., on the application of the Collegiate System to the Medical Schools of the Metropolis, by the Rev. J. H. North, M.A. Chaplain to St. George's Hospital, London, 1841, 8vo, pp. 15.

Such being the state of the medical pupil,—or at least of the majority,—during his residence in London, "a sheep having no shepherd," can it be wondered at that his good intentions speedily give way to the seductive allurements of vicious companions? His moral and religious principles are injured, perhaps destroyed. Cards, billiards, and every sort of vicious incentive lead him on; his studies are neglected; his time, too short even under the best system of economy, is wasted, and he at last contents himself with the disgraceful alternative of cramming his memory, under guidance of a Grinder, just up to that point which may cheat the vigilance of the examiners. And thus he leaves the hospitals to engage in practice, but very little wiser than he entered them, having completely wasted the most valuable years of his educational life entirely from having begun badly, from never having had a fair start in his course.

It may be asserted that the picture we have now drawn is exaggerated, and appeals may be made on the opposite side to the examination lists of every year which are published by every school, as proofs that under the present system the students are not so lost to a sense of their duty, but have shown themselves eminently distinguished both morally and intellectually; while the numerous distinguished men of whom our profession boasts, both in town and country, may be adduced as further proof that the present system has not worked amiss. It may be further stated in extenuation of the present practice, that in few of the continental universities is there any collegiate system of restraint adopted, while we all know that the same may be said of Edinburgh and all the Scotch universities, as well as of the university of Dublin.

To these arguments we answer, first, that we have not pretended that the sketch above drawn includes every one who enters to the schools. Allowing that all, and more than all, who receive honorable public approval have well deserved it, there still remains a large proportion among whom the seeds of vice and folly, unchecked by a judicious system of training, may and do grow and fructify to a fearful extent. That the examples which do credit to their profession are as numerous as they are, may mainly be attributed to the exertions and the example of the present race of professors. We do not hesitate to express our conviction, that their diligence and their right feeling have, as a body, done much, perhaps as much as is possible under the present system, to remedy the evils complained of. But this is not enough: a large and most influential body of the community must not be permitted to encounter in their very outset dangers which may materially injure and impair their future usefulness, and may render them rather a curse than a blessing to that community.

As to the state of Foreign universities or even of those in our own country, we must first be much more certain than we at present are, that they would not, one and all of them, be much benefited and improved by the adoption of some such system as we now advocate, before we can allow much weight to any argument drawn from their practice. On this point we cannot forbear giving the words of a man of no mean authority, Dr. Pusey, as quoted in an excellent article in the Quarterly Review for Jan. 1840. Speaking of the German universities Dr. Pusey says, "On the removal of the student to the university he passes at once from boyhood to manhood; at once, instead of discipline and control, he is left almost unfettered even by moral guidance; the only requisition made is, that he should attend one or more sets of lectures. Some general advice is also given him as to the method which it may be most advantageous for him to pursue; but beyond this, what instruction he should receive, and from whom, whether he should live as a Christian or as a heathen (provided he interrupt not the public peace) is left to his own option." In this description of the errors of the German schools we at once recognize those of our own, and so correct is the likeness that we might with slight alteration substitute the one for the other. But even granting that the discipline in foreign schools is just what it should be, the condition and the requirements of a London university must widely differ on this point from any or all of them. The vast size of our metropolis has, we doubt not, a very prejudicial effect upon the moral habits of a class of young men thrown together without any feeling of responsibility or control. Indeed we cannot but consider that this fact alone, in the present state of things, constitutes a very decided and important objection to the metropolis as the site of medical schools; and we rejoice, on this ground, at the recent establishment of so many excellent medical schools in the provinces. And though the eminence of the metropolitan professors, and the numerous advantages possessed by the London schools will always attract the greater part of those entering the profession, yet we must look upon an education in the country as having at least superior advantage in a moral point of view, and as thus possessing the power to abate, in some degree, the evil which we now hope to see abated in London.

Let us now examine somewhat more in detail the system which is at present adopted by students in London, and enquire what are the facilities for the introduction of a complete plan of collegiate discipline. We fear that unvarnished truth must declare, that from beginning to end, whether with regard to moral discipline or professional study, system there is none. The only guide as to the course which the student is to take is his own inexperienced judgment, assisted by the curriculum of required attendance upon lectures from the Apothecaries' Hall and the College of Surgeons; books he is to select at his own discretion, and the disposal of his time is under the same direction. On first coming to the London hospitals, one of two plans is generally adopted; either he is placed in the family of some surgeon or physician in the neighbourhood of the school that he is attending, or, which is much the most frequent plan, he takes a lodging in the vicinity, generally in the same house with two or three other students. In this last case, of course, the time and conduct of these gentlemen is under no control whatever, with the exception of whatever influence the particular professors may have in their intercourse with them in the school, which is necessarily of a very limited extent. The only exception to this general rule is to be found in King's College. The late excellent principal, Mr. Rose, had much at heart the introduction of collegiate discipline in that school, and his views have received encouragement and support from all the professors,* but hitherto their practical application has been very limited. Sufficient however has been done to convince all who have observed the influence of this arrangement, of the great advantages to be derived from it, and nothing now seems wanting but the energetic efforts of the friends of that institution, to carry out the desired plan with complete effect.† All the other colleges and schools have confessedly made no attempt to secure any further influence over the students than what may be effected by the general system of annual rewards for the most industrious, and the very occasional correction of the most idle by the withholding of their certificates of attendance upon lectures.

The universities of Oxford and Cambridge offer to us the only models for a complete system of collegiate superintendence. It is not professed that the discipline is here so complete that idleness is eschewed, and vice entirely kept in check; indeed whatever advantages the system may offer, we are aware that its rules are most imperfectly carried out even in these favoured spots, and therefore the amount of good which is actually produced may be presumed to be only a part of what might be expected from the more perfect administration of more perfect laws.

It is unnecessary to enter into any details respecting collegiate arrangements so well known as those of our ancient universities. We shall pass in review their more characteristic features, and consider the adaptation of a modified system of the same general kind to the wants of the London schools.

1. *College Rooms.* We are quite aware that there are objections to the crowding

* See British Magazine for January, 1840, for some observations on the enlarging the accommodation for medical students at King's College, by Professor Todd.

† The rooms at present provided for students in King's College form, it must be remembered, but a small part of the plan for improvement of the condition of the students which we contemplate. There is little or no control exercised over their time or their pursuits, and there is no system of college tutorage attempted. We must not therefore take this feeble attempt as any sample either of the advantages of the college system or of the facility of its adoption.

together a number of young men, and that the fears of some sincere friends to the introduction of a better system than the present, are strong upon this head. We confess however that, knowing as we do the difficulties which require to be overcome in the introduction of a new system—and that one which must to a certain degree curtail the liberty of young men—we still are so certain of the advantages which must arise from a *judicious* and *cautious* administration of the discipline, that we ourselves have no fears whatever as to its ultimate success. There is one principle, however, in connexion with this part of the question, which must, at least at first, be admitted, to meet the prejudices and the jealousy of control, natural at the age of medical students. We should recommend, that for the present the submission to the restraints of college life should be made *optional* with the student. The advantages which are certain to flow from it must be the inducement to him to submit to its restraints, and if for any reason he prefers being left entirely at liberty, let him have his will. By this means one great advantage will result to those within the college, that the dissipated and ill disposed will be separated from them, and thus a great hindrance to their studies and good conduct will be removed. There are also many obvious and sufficient reasons which would induce some of even the best disposed to prefer living at a distance from the hospital; as, for instance, in the case of the residence of the pupil in the house of his parents, or their relations, or friends.

In reference to the possibility of providing accommodation for all that may require it, we request attention to the following considerations. The average cost to the medical pupil of his lodging at present is about 14*s.* per week, which, granting that he stays only eight months in the year, makes £24. For this sum he is often content to obtain a small bed-room and sitting-room, in a confined gloomy situation. Now, if we fix the average terms to be demanded by the college at £20,* one hundred resident students would produce an income of £2000. Out of this deduct £500 for incidental current expenses, such as servants' wages, repairs, &c. &c., and we have still £1500 as the return for the capital invested in the building. This sum, calculated at 5 per cent. would be the equivalent of £30,000 principal, a sum, we conceive, more than sufficient for the required purpose. But it is to be borne in mind, that this calculation is made at a high rate of interest, certainly higher than money might be raised for; not to mention all abatement on the score of donations, which would certainly be received towards so good a cause. We have also here provided only for 100 students, but few of the large schools would have so small a number accommodated within their walls, and some would have many more: moreover, the larger the building the less proportionally will be the expense;—that is, room for 200 pupils would not cost the double of what would be required for 100. The *average* rate of charge to the student might, as we have said, be £20; but, of course, this must vary in each case according as he chose to have one or two rooms, or according to the floor on which he lived, as in the chambers at the inns of court.

If a college residence be thus provided for the students, it is obvious that a system of regular discipline in regard to hours of admission at the gates must be adopted to give the whole system that efficient power of control which is essential to the proper governing and guiding of a body of young men. We do not, however, say more upon a part of the subject on which we may be opposed to the views of many well wishers to the collegiate system, because we now only desire to make a general sketch of our proposed plan. The details may be further discussed hereafter. We now only desire to give an impulse to the public feeling already excited.

Though it will be highly desirable that all students who are willing should be received within the walls of the building, yet under some circumstances of want of accommodation, licensed lodging-houses might be permitted, under the same rules and discipline as in the college itself; but of course all freshmen, and as

* The rooms at King's College are let at from £25 to £30 for the whole year. It is desirable to charge these as low as possible, as a great number of students would be prevented from entering them by inability to pay at so high a rate as this.

many as possible of the senior students, should be required to live within the walls.

2. *Tutors.* The establishment of resident college tutors is the most important part of all the system under discussion. This is an improvement that is imperatively demanded, and which is so reasonable, and so easily accomplished, that if nothing else is done this must. At Cambridge and Oxford,* (where, by the bye, the college tutor is by no means what he might be and ought to be,) this functionary is appointed from among the fellows, and receives from each pupil allotted to him from £2. 10s. to £4 per quarter; or from £10 to £16 a year. If twenty-five students are appointed to be under his tuition, this will realize to him an income of £250 to £400. At the Uuiversities the tutors have also their fellowships, and these, together with the pupils' fees, produce a handsome income. This however is not to be expected or indeed desired in London. Let a tutor be appointed to every twenty-five students who shall pay him each £8 or £10 a year: this will give him an income of £200 or £250; and of course his rooms will be found for him, and perhaps his board. These tutors might be chosen by examination from the most meritorious of the third years' pupils; and though some preference should be shown to the members of the particular school, we should advise that the election should not be restricted to them, but thrown open to all the schools. How many men of superior talents and attainments would be delighted to obtain an appointment, which would enable them to pursue their studies with comfort for several years longer than they might otherwise be able to do! The duties of the tutor should be much the same as they are at the Universities. He would be the friend and guide of the pupil; his Mentor in every respect. He would superintend and assist his studies, practical and theoretical, and second the instructions of the Professors by frequent catechetical exercises. His superintendence should not be permitted to extend to more than the number we have mentioned, lest its efficiency might be proportionately diminished. By means of this system of tutelage, the student would be saved from vast waste of time and labour, and at a less expense than he now pays for a three-months' assistance from the *grinder*, that dernier resort of idleness. This disgraceful system of pretended instruction we should hope to see entirely superseded by the proposed reform. If it should be thought unadvisable that the tutors should be chosen out of the class of students, and that the office would require men of longer standing to keep up due authority, we have no doubt that such an office would produce numerous candidates. There are many most competent persons who would prefer such a life to the anxieties of private practice. Perhaps a beneficial arrangement might be—for the tutors of the first-year's-men to be chosen according to the first plan; and the two-years'-men might have these senior tutors.

We feel confident, from a considerable experience among hospital pupils, that a plan of instruction, such as might be carried on somewhat according to the above suggestions, would so exactly accord with the wants and wishes of this class of young men, that this alone would be a sufficient inducement to them to submit to the partial restraints necessary in a college life.† Additional inducements might be found in privileges, rewards, and appointments, to be competed for only by those within the rules.

3. *Domestic arrangements.* There should be one or two dining halls in the college. When the numbers are large we should prefer two. The students might be classed as first and second-years'-men, and occupy different parts of the building accordingly, and use different dining halls; the tutors always dining with their classes.

4. *Religious observances.* Prayers should be read at the chapel of the college night and morning, and encouragement given to the regular attendance on them

* In all our statements of the system pursued at those Universities we have not studied minute accuracy: indeed there are peculiarities and differences in each, but a general view of the facts is all that is required for our purpose.

† In King's College and University College the general classes might partake of the privileges of the college rooms, and tutors might be appointed for them upon the same principles as those for the medical classes.

at least once on every day, and not less than once on Sunday. How far it might be desirable to adopt the system of fines for non-attendance, we have strong doubts; and we have not space, nor is it at present necessary to discuss the point. Of this, however, we are certain, that the regular attendance upon the ordinances of religion as a means of moral control, as well as of religious instruction, is of the greatest importance to the younger members of our profession, who in the exercise of that profession are about to enter upon duties of great moral responsibility and difficulty, and who therefore peculiarly require the guiding influences of a religious and moral education.

5. *Costume.* A particular academical dress is a matter of much less importance in London than in Oxford and Cambridge; and we have even doubts of its utility: it certainly is not necessary.

6. *Rewards and Penalties.* It may be objected that a system of Collegiate discipline must be incomplete without the adoption of rewards and penalties for the enforcement of the authority of the College, and that there will be serious difficulties opposed to the exercising of such authority unless all the metropolitan schools come in to the same plan. We answer that to a judicious and cautious application of such control there will be no difficulty on the part of the student, for whose benefit it is to be adopted, who will cheerfully submit to a necessary control for the sake of the immense benefits to be palpably derived from the whole system. Neither need the individual school which shall boldly put itself forward to remedy the present evils fear to stand alone, since the object in view is confessedly so important that there can be no doubt that the existing examining bodies must and will gladly assist the undertaking by their sanction and support, and by granting a proper degree of consideration and weight at their examinations to the certificates of good conduct accorded by the individual college. It will obviously be to the interest of the school to temper and soften the necessary strictness of its rules, and to encourage submission to them by rewarding good conduct.*

On reviewing the brief and imperfect survey which we have made, both of the evil and the remedy, we feel confident that we have overstated neither the magnitude of the one, nor the feasibility of the other. Deeply impressed as we are with the malignancy of the disease, we are satisfied that, if by any hint of ours we may have contributed towards the successful application of the remedy, we shall have rendered a high and most important service to our profession and, through its members, to the public. We rejoice, indeed, to see that the community at large are at length becoming sensible of the important and inseparable connexion between its interests and ours. The moral as well as the intellectual character of our profession must necessarily have a most important bearing upon the welfare and happiness of society in general. On every account, therefore, we hope most earnestly that the governors of the metropolitan hospitals, and the lay patrons and supporters of our schools, will feel that it is their bounden duty—assuredly it is their interest—now to use their best efforts, in conjunction with their professors and medical officers, to organize an efficient system of collegiate discipline for the benefit of their pupils. Indeed, according to our own opinion, the subject rightly viewed can leave no option to those who have the management and control of our medical institutions. They are bound by all the sanctions of morality and of religion no longer to allow a large body of our youth to lie exposed to the uncontrolled temptations of a vicious metropolis. Of the success of the proposed plan, if once set on foot, we are so confident that we venture to prophesy that if only one of our schools sets the example, the rest must, in self-defence, follow; and nothing would gratify us more sincerely than to witness the commencement of this honorable rivalry so pregnant with advantage to our profession.

* Might not a large establishment of the kind in question afford facilities for the establishment of scholarships by which, at a slight expense to the institution, board and lodging or rooms only might be given as a prize to the most deserving students for one or two years?

